# Oleanolic Acid Attenuates Insulin Resistance via NF-*κ*B to Regulate the IRS1-GLUT4 Pathway in HepG2 Cells

**DOI:** 10.1155/2015/643102

**Published:** 2015-12-30

**Authors:** Ming Li, Zongyu Han, Weijian Bei, Xianglu Rong, Jiao Guo, Xuguang Hu

**Affiliations:** Key Unit of Modulating Liver to Treat Hyperlipemia SATCM (State Administration of Traditional Chinese Medicine), Level 3 Lab of Lipid Metabolism SATCM, Guangdong TCM Key Laboratory for Metabolic Diseases, Guangdong Pharmaceutical University, Guangzhou Higher Education Mega Centre, Guangzhou 510006, China

## Abstract

The aim of our study is to elucidate the mechanisms of oleanolic acid (OA) on insulin resistance (IR) in HepG2 cells. HepG2 cells were induced with FFA as the insulin resistance model and were treated with OA. Then the glucose content and the levels of tumor necrosis factor-*α* (TNF-*α*) and interleukin-6 (IL-6) were analyzed. Moreover, protein expression of nuclear factor kappa B (NF-*κ*B), insulin receptor substrate 1(IRS1), and glucose transporter 4 (GLUT4) in cells treated with OA were measured by Western blot analysis. Additionally, IRS1 protein expression exposed to OA was detected after using pyrrolidine dithiocarbamate (PDTC).Our results revealed that OA decreased the glucose content in HepG2 cells in vitro. Moreover, OA reduced the levels of TNF-*α* and IL-6 and upregulated IRS1 and GLUT4 protein expression. Furthermore, OA also reduced NF-*κ*B protein expression in insulin-resistant HepG2 cells. After blocking NF-*κ*B, the expression of IRS1 protein had no obvious changes when treated with OA. OA attenuated insulin resistance and decreased the levels of TNF-*α* and IL-6. Meanwhile, OA decreased NF-*κ*B protein expression and upregulated IRS1 and GLUT4 protein expression. Therefore, regulating the IRS1-GLUT4 pathway via NF-*κ*B was the underlying mechanism of OA on insulin resistance.

## 1. Introduction

Most pathology ultimately arises from obesity's characteristic milieu of chronic low-grade inflammation and insulin resistance [1]. The inability of insulin to perform normal biological functions in vivo is called insulin resistance. In general, insulin resistance occurs when a certain concentration of insulin cannot effectively stimulate glucose uptake and utilization in the peripheral target organs. Thus the organism suffers from impaired glucose tolerance that ultimately leads to diabetes or other diseases [2–4]. Many studies have demonstrated that patients with insulin resistance (IR) have displayed an increased risk of developing diabetes, cardiovascular disease, and other diseases [5, 6].

**Figure 1:**
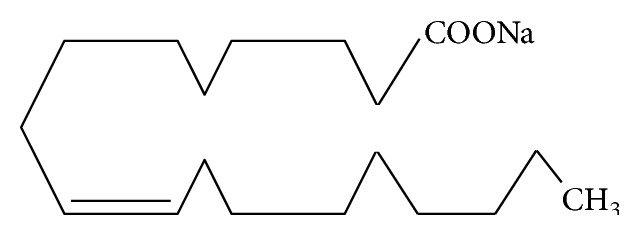
Chemical structure of sodium oleate.

Recent studies have found that metabolic diseases including obesity are a common cause of insulin resistance. Meanwhile, increases in FFA (sodium oleate, Figure 1) levels have been shown to occur in metabolic diseases [7–9]. Higher FFA levels could induce the body to secrete inflammatory cytokines, such as TNF-*α* or IL-6, which would be a low-grade inflammatory state. Inflammation induced by FFA plays a key role in insulin resistance [10, 11]. As the important target of the inflammatory pathways, NF-*κ*B could disrupt IRS1 and downregulate the expression of IRS1 under conditions of insulin resistance [12, 13].

**Figure 2:**
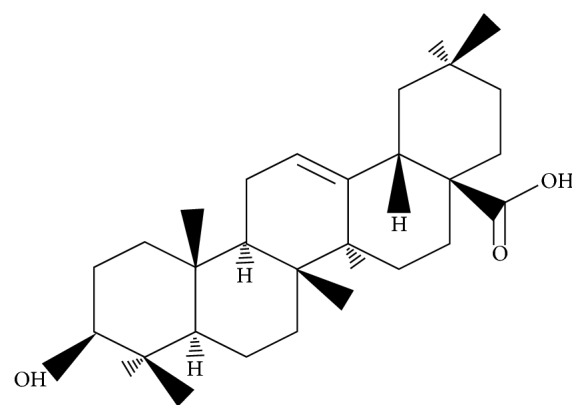
Chemical structure of oleanolic acid.

Oleanolic acid (OA), a naturally occurring triterpenoid (Figure 2), is the main effective active ingredient in many herbs such as glossy privet fruit and exists largely in food products (vegetable oils) [14].

Preliminary study found that OA had blood glucose-reducing effect and inhibited insulin resistance in diabetic rats [15]. OA decreased blood glucose, improved insulin resistance, and enhanced insulin signaling by inhibition of ROS and anti-inflammatory effect in diabetic mice [16]. OA also regulated the NF-*κ*B signaling which exhibits high anti-inflammatory activity and was regarded as a potential NF-*κ*B inhibitor [17]. Recently a study conducted by Li et al. demonstrated that OA ameliorated insulin resistance via the IRS-1/PI3k/Akt pathway in rats [18]. Although previous evidence showed that OA attenuated insulin resistance in part through inhibiting inflammation and enhancing the IRS-1 signal, the role of NF-*κ*B in attenuating insulin resistance by OA remains essentially unknown.

To further elucidate the molecular mechanisms of OA on insulin resistance and investigate the role of NF-*κ*B in regulating IRS-1 signal by OA, the levels of TNF-*α* and IL-6 were analyzed and protein expression of nuclear factor kappa B (NF-*κ*B), insulin receptor substrate 1 (IRS1), and glucose transporter 4 (GLUT4) in insulin-resistant HepG2 cells treated with OA was measured. In addition, IRS1 protein expression exposed to OA was detected after NF-*κ*B was blocked using pyrrolidine dithiocarbamate (PDTC).

## 2. Methods and Materials

HepG2 cells were purchased from Landbiology (Guangzhou, China, lot: HB-8065). Dulbecco's modified Eagle's medium (DMEM) was bought from GIBCO (Gibco, Grand Island, NY, USA, lot: 8114176). Fetal bovine serum (FBS) was purchased from Biological Industries (Israel, lot: 1415878). NF-*κ*B, IRS1, and GLUT4 antibodies were from Abcam Inc. (Cambridge, UK, lot: GR165665-1; GR95405-9; GR56566-1). Rosiglitazone (RSG) was bought from Sigma (St. Louis, MO, USA, lot: R2408-10 mg). Sodium oleate was bought from Tokyo Chemical Industry (Tokyo, Japan, lot: W76EC-0J). All other reagents were analytical grade. A GOD-POD kit was purchased from Biosino Bio-Technology and Science Inc. (Beijing, China, lot: 143271). ELISA kits were bought from Raybiotech Inc. (Norcross, GA, USA, Human TNF-*α*, lot: 0926140193; human IL-6, lot: 0926140140). Oleanolic acid (OA) was purchased from the National Institutes for Food and Drug Control (Beijing, China, lot: 110709-200505).

### 2.1. Cell Culture

The human hepatocellular carcinoma cell line HepG2 was purchased from Land Unicomed. Cells were cultured in DMEM supplemented with 10% heat-inactivated FBS at 37°C in a 5% CO_2_ atmosphere. In all experiments, the cells were grown to 80–90% confluence.

### 2.2. Cell Viability Assay

The cytotoxicity of OA against the HepG2 cells was assessed by the MTT assay. The MTT viability assay was described previously [19]. Briefly, HepG2 cells were plated in 96-well plates at 1 × 10^5^ cells per well. After 24 h, HepG2 cells were treated with indicated dose of OA at 37°C for 24 h; MTT stock solution (20 *μ*L; 5 mg/mL in PBS) was added to each well to achieve a total reaction volume of 220 *μ*L. After 4 h of incubation at 37°C and 5% CO_2_, the media were then removed and 150 *μ*L dimethyl sulfoxide (DMSO) was added to every well. After shaking for 10 min, the amount of purple formazan was assessed by measuring the absorbance at 490 nm.

### 2.3. Induction of Insulin Resistance in HepG2 Cells and Glucose Utilization Experiments

The HepG2 cells were cultured and determination of glucose utilization was performed as previously described [20]. Briefly, HepG2 cells were seeded on 24-well plates at 1 × 10^5^ cells/well and incubated for 24 h to reach maximal confluence. The cells were then incubated for 24 h in serum-free DMEM, 0.2% BSA, and 200 *μ*mol/L sodium oleate in the absence or presence of OA (OA was dissolved in DMSO) or RSG. Next, cells were washed twice with PBS and incubated for 3 h in serum-free DMEM containing 25 mmol/L d-glucose and 1 × 10^−9 ^mol/L insulin. The culture medium was collected. The content of glucose was quantified using a GOD-POD kit.

### 2.4. Enzyme-Linked Immunosorbent Assay of TNF-*α* and IL-6 Levels

Insulin resistance was induced in HepG2 cells as previously described. The culture medium was centrifuged at 14000 ×g for 10 minutes at 4°C. The supernatant was then collected and stored at −80°C until analysis. The levels of TNF-*α* and IL-6 in the supernatant were determined using ELISA kits according to the manufacturer's instructions.

### 2.5. Western Blot Analysis

Insulin resistance was induced in HepG2 cells as previously described. Cells were washed with ice-cold PBS and lysed with a RIPA lysis buffer. For Western blotting, protein samples (20 *μ*g) of sodium oleate induced insulin-resistant HepG2 cells were separated via 10% sodium dodecyl sulfate-polyacrylamide gel electrophoresis (SDS-PAGE). The proteins were transferred to a PVDF membrane and incubated with primary antibody (anti-NF-*κ*B, anti-IRS1, anti-GLUT4, or anti-GAPDH), followed by a secondary antibody (horseradish peroxidase-conjugated anti-rabbit IgG). The intensity of the immunoblot signal was assayed using Western Bright ECL spray and analyzed quantitatively using GeneTools software from Syngene (Syngene, Cambridge, UK).

To choose the most effective concentration of PDTC, the cells were incubated for 26 h in serum-free DMEM at the different dosages of PDTC or 0.2% BSA and 200 *μ*mol/L sodium oleate. The protein samples were prepared for Western blot experiments and incubated with anti-NF-*κ*B.

Then, the cells were incubated for 2 h in serum-free DMEM and PDTC with the most effective concentration (all PDTC groups). After that, 0.2% BSA and 200 *μ*mol/L sodium oleate and three dosages of OA were added. Next, the cells were incubated for an additional 24 h after preparing the protein samples in Western blot experiments and incubated with anti-IRS1.

### 2.6. Statistics Analysis

The statistical analyses were conducted using SPSS16.0 software. All results are presented as the mean ± standard deviation (SD). Statistical analyses were performed using analysis of variance (one-way ANOVA) followed by the Student-Newman-Keuls test for significance. The differences were considered to be statistically significant at *P* < 0.05.

## 3. Result

### 3.1. Influence of OA on the Cell Viability in HepG2 Cells

**Figure 3:**
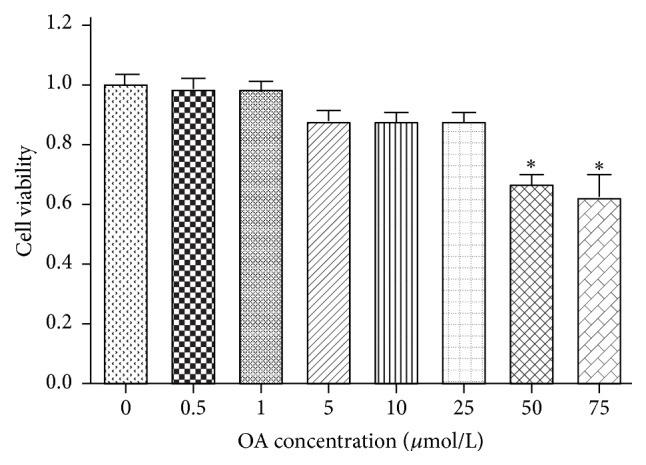
Influence of OA on the cell viability in HepG2 cells. 1 × 10^5^ cells per well were seeded into a 96-well plate; cells were stimulated with various concentrations of OA (1–75 *μ*mol L^−1^) for 24 h; cell viability was measured by MTT. ^*∗*^ *P* < 0.05.

The influence of OA on the cell viability in HepG2 cells was examined using the MTT reduction assay. The MTT assay results demonstrated no significant difference in cell viability of cells treated with OA at the concentrations of lower than 50 *μ*mol/L. As the concentration of OA was 75 *μ*mol/L, the cell viability was reduced by approximately 40% when compared to the control treatment (Figure 3).

### 3.2. Effect of OA on the Glucose Content of the Culture Media in Insulin-Resistant HepG2 Cells

**Figure 4:**
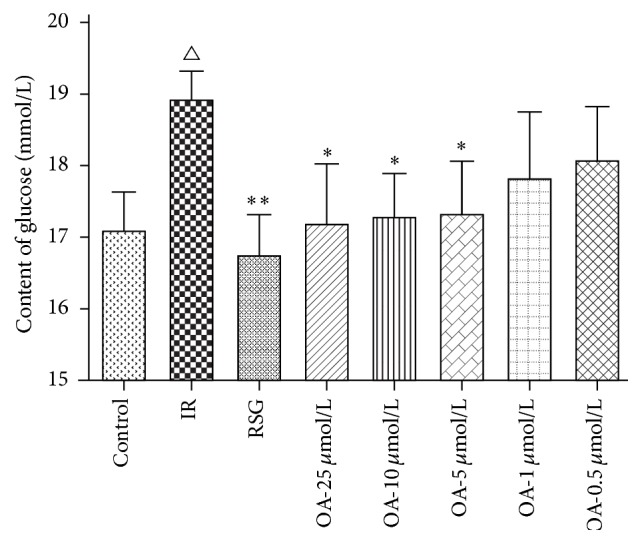
Effect of OA on the glucose content of the culture media in HepG2 cells. ^△^ *P* < 0.01 compared with the control cells; ^*∗∗*^ *P* < 0.01, ^*∗*^ *P* < 0.05 compared with the IR cells.

The HepG2 cells were incubated for 24 h in serum-free DMEM containing 200 *μ*mol/L sodium oleate, in either 0.5, 1, 5, 10, and 25 *μ*mol/L OA or RSG (10 *μ*mol/L). Next, the cells were incubated for 3 h in insulin. The glucose content in the insulin-resistant HepG2 cells in culture medium was significantly increased compared with the control cells (*P* < 0.01). After treatment with OA (5, 10, and 25 *μ*mol/L), the glucose content in the culture medium significantly reduced compared with the IR cells (*P* < 0.05). Rosiglitazone (RSG), which is the insulin sensitizer as a positive control drug, decreased the glucose content in the culture medium compared with the IR cells (*P* < 0.01). These results showed that OA attenuated insulin resistance in a dose-dependent manner (Figure 4).

### 3.3. Effect of OA on the Levels of TNF-*α* and IL-6 in Insulin-Resistant HepG2 Cells

**Figure 5:**
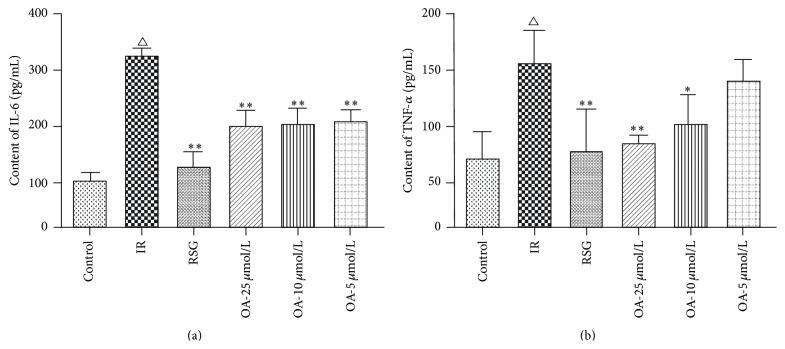
Effect of OA on TNF-*α* and IL-6 levels in HepG2 cells. ^△^ *P* < 0.01 compared with the control cells; ^*∗∗*^ *P* < 0.01, ^*∗*^ *P* < 0.05 compared with the IR cells.

To examine the effect of OA on inflammatory cytokines, the levels of TNF-*α* and IL-6 in the culture media of insulin-resistance HepG2 cells were measured by ELISA. The HepG2 cells were incubated for 24 h in serum-free DMEM containing 200 *μ*mol/L sodium oleate, in the 5, 10, and 25 *μ*mol/L OA, or in RSG (10 *μ*mol/L). The levels of TNF-*α* and IL-6 in insulin-resistant HepG2 cells were significantly higher compared with the control cells (*P* < 0.01). After treatment with OA (5, 10, and 25 *μ*mol/L), the level of IL-6 in the culture medium was significantly lower compared with the IR cells (*P* < 0.01). Moreover, treatment with OA at the dosages of 10 and 25 *μ*mol/L significantly lowered the levels of TNF-*α* in the culture medium compared with the IR cells (*P* < 0.05; *P* < 0.01). Moreover, after treatment with OA at a dosage of 5 *μ*mol/L, the level of TNF-*α* in the culture medium did not obviously decrease compared with the IR cells. RSG was also able to decrease all of the levels of TNF-*α* and IL-6 in the culture medium compared with the IR cells (*P* < 0.01) (Figure 5).

### 3.4. Effect of OA on the Protein Expression of NF-*κ*B, IRS1, and GLUT4 in Insulin-Resistant HepG2 Cells

**Figure 6:**
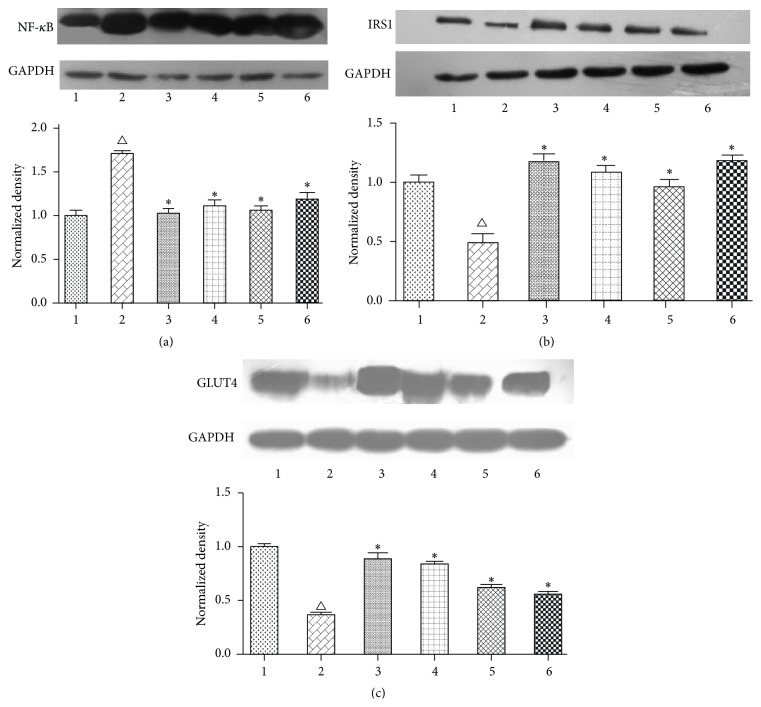
Effect of OA on NF-*κ*B, IRS1, and GLUT4 protein expression. (1) Control; (2) IR; (3) RSG; (4) OA-25 *μ*mol/L; (5) OA-10 *μ*mol/L; (6) OA-5 *μ*mol/L. The protein expression of NF-*κ*B, IRS1, and GLUT4 was measured via Western blotting as described in the text. The figures represent one of three experiments with similar results. ^△^ *P* < 0.01 compared with the Control group; ^*∗*^ *P* < 0.01 compared with the IR group.

To elucidate the mechanism of OA on insulin resistance in HepG2 cell, a Western blot analysis was used to measure the protein expression of NF-*κ*B, IRS1, and GLUT4 in HepG2 cells. The cells were divided into six groups, including a Control group, IR group (200 *μ*mol/L sodium oleate), RSG group (200 *μ*mol/L sodium oleate and RSG 10 *μ*mol/L), OA-25 *μ*mol/L group (200 *μ*mol/L sodium oleate, OA 25 *μ*mol/L), OA-10 *μ*mol/L group (200 *μ*mol/L sodium oleate, OA 10 *μ*mol/L), and OA-5 *μ*mol/L group (200 *μ*mol/L sodium oleate, OA 5 *μ*mol/L). As shown in Figure 4, the expression of NF-*κ*B protein in the IR group was significantly greater than the Control group (*P* < 0.01). IRS1 and GLUT4 protein expression in the IR group was significantly reduced compared with the Control group (*P* < 0.01). After treatment with OA at three dosages, the protein expression of NF-*κ*B was significantly reduced compared with the IR group (*P* < 0.01). In addition, the expression of IRS1 and GLUT4 protein with OA at three dosages was significantly higher compared with the IR group (*P* < 0.01). RSG was also able to increase the expression of IRS1 and GLUT4 protein and reduce the expression of NF-*κ*B protein compared with the IR group (*P* < 0.01) (Figure 6).

### 3.5. Effect of PDTC on NF-*κ*B Protein Expression in HepG2 Cells

**Figure 7:**
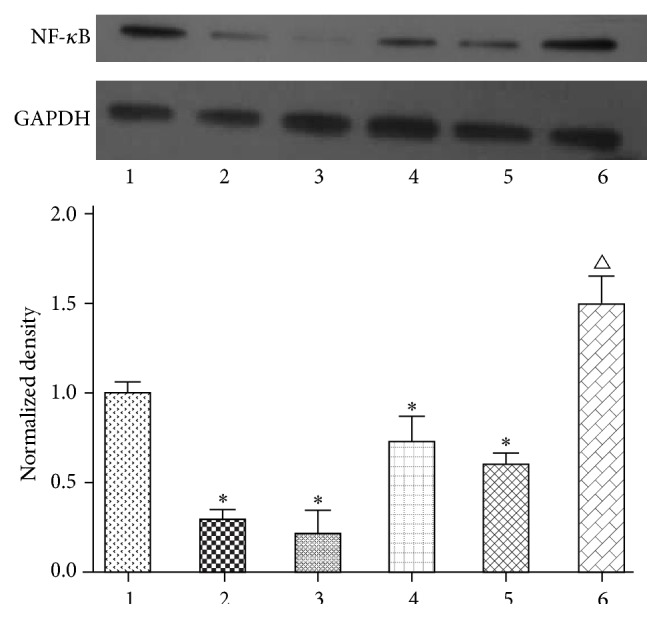
Effect of PDTC on NF-*κ*B protein expression. (1) Control; (2) PDTC-100 *μ*mol/L; (3) PDTC-300 *μ*mol/L; (4) PDTC-500 *μ*mol/L; (5) PDTC-1000 *μ*mol/L; (6) IR. The protein expression of NF-*κ*B was measured via Western blotting as described in the text. The figures represent one of three experiments with similar results. ^△^ *P* < 0.01 compared with the Control group; ^*∗*^ *P* < 0.01 compared with the Control group.

To select the most effective concentration of PDTC that blocked NF-*κ*B expression using Western blot analysis, HepG2 cells were divided into six groups, including a Control group, 100 *μ*mol/L PDTC group, 300 *μ*mol/L PDTC group, 500 *μ*mol/L PDTC group, 1000 *μ*mol/L PDTC group, and IR group (200 *μ*mol/L sodium oleate). The levels of NF-*κ*B expression in all groups that were treated with PDTC were significantly lower than the Control group (*P* < 0.01). The expression of NF-*κ*B in the IR group was significantly greater than the Control group (*P* < 0.01). As shown in Figure 5, the most effective concentration of PDTC that blocked NF-*κ*B expression was 300 *μ*mol/L (Figure 7).

### 3.6. Effect of OA on the Expression of IRS1 Protein in Insulin-Resistant HepG2 Cells with Blocking of the Expression of NF-*κ*B

**Figure 8:**
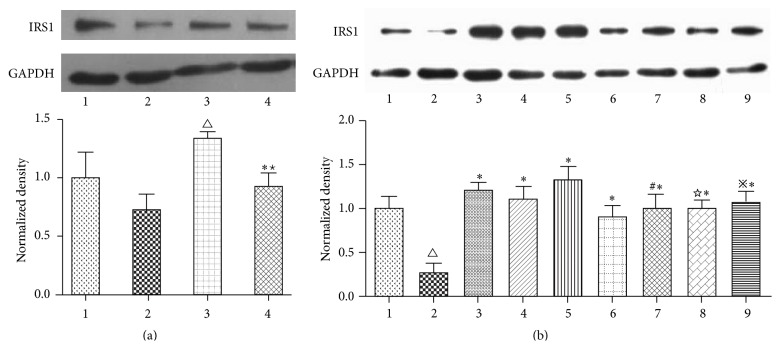
Effect of OA on IRS1 protein expression with blocking of the expression of NF-*κ*B. (a) (1) Control; (2) IR; (3) P + Control; (4) P + IR. (b) (1) Control; (2) IR; (3) OA-25 *μ*mol/L; (4) OA-10 *μ*mol/L; (5) OA-5 *μ*mol/L; (6) P + IR; (7) P + OA-25 *μ*mol/L; (8) P + OA-10 *μ*mol/L; (9) P + OA-5 *μ*mol/L. The protein expression of IRS1 was measured via Western blotting as described in the text. The figures represent one of three experiments with similar results. ^△^ *P* < 0.01 compared with the Control group; ^*∗*^ *P* < 0.01 compared with the IR group; ^*^ *P* < 0.05 compared with the P + Control group; ^#^ *P* < 0.05 compared with the OA-25 *μ*mol/L group; ^☆^ *P* < 0.05 compared with the OA-10 *μ*mol/L group; ^*※*^ *P* < 0.05 compared with the OA-5 *μ*mol/L group.

*Figure 8(a)*. To study the IRS1 protein expression when PDTC exists or not, HepG2 protein samples were divided into four groups as follows: Control group, IR group (200 *μ*mol/L sodium oleate), P + Control group (300 *μ*mol/L PDTC), and P + IR group (300 *μ*mol/L PDTC and 200 *μ*mol/L sodium oleate). As shown in Figure 6(a), the expression of IRS1 protein in the P + Control group was significantly greater than the Control group (*P* < 0.01). In addition, the expression of IRS1 protein in P + IR group was significantly increased compared with the IR group (*P* < 0.01) and significantly reduced compared with the P + Control group (*P* < 0.05).

*Figure 8(b)*. To evaluate the effect of OA on the IRS1 protein expression when PDTC exists or not, HepG2 protein samples were divided into nine groups as follows: Control group, IR group (200 *μ*mol/L sodium oleate), OA-25 *μ*mol/L group (200 *μ*mol/L sodium oleate, 25 *μ*mol/L OA), OA-10 *μ*mol/L group (200 *μ*mol/L sodium oleate, 10 *μ*mol/L OA), OA-5 *μ*mol/L group (200 *μ*mol/L sodium oleate, 5 *μ*mol/L OA), P + IR group (300 *μ*mol/L PDTC, 200 *μ*mol/L sodium oleate), P + OA-25 *μ*mol/L group (300 *μ*mol/L PDTC, 200 *μ*mol/L sodium oleate and 25 *μ*mol/L OA), P + OA-10 *μ*mol/L group (300 *μ*mol/L PDTC, 200 *μ*mol/L sodium oleate and 10 *μ*mol/L OA), and P + OA-5 *μ*mol/L group (300 *μ*mol/L PDTC, 200 *μ*mol/L sodium oleate and 5 *μ*mol/L OA). As shown in Figure 6(b), the expression of IRS1 protein in the IR group was significantly lower than the Control group (*P* < 0.01); the expression of IRS1 protein in the OA-25 *μ*mol/L group, OA-10 *μ*mol/L group, OA-5 *μ*mol/L group, P + IR group, P + OA-25 *μ*mol/L group, P + OA-10 *μ*mol/L group, and P + OA-5 *μ*mol/L group was significantly greater than the IR group (*P* < 0.01). When compared to the three dosages of OA without PDTC, the expression of IRS1 protein in three dosages of OA with PDTC was reduced (*P* < 0.05) (Figure 8).

## 4. Discussion

This study demonstrated that OA attenuated insulin resistance in HepG2 cells, whose effect is possibly mediated through decreasing the levels of TNF-*α* and IL-6 and regulating the expression of IRS1 and GLUT4 protein via the NF-*κ*B protein.

Elevated levels of free fatty acids are thought to be the pathogenic factors causing metabolic disorders such as obesity and diabetes [21]. Oleate, an unsaturated fatty acid, can induce insulin resistance in HepG2 cells [22]. HepG2 cells were wildly used for studying insulin resistance [23]. When HepG2 cells were induced with insulin resistance, their responses to glucose were affected. The glucose content in insulin-resistant cells in culture medium increased compared with healthy cells [24]. In the present study, we used 200 *μ*mol/L sodium oleate to induce insulin resistance in HepG2 cells. The glucose content in the IR group in culture medium was significantly greater than the Control groups. It showed that it is a suitable model of insulin resistance.

Oleanolic acid (OA) exerts multiple pharmacological actions including glycoregulatory, hepatoprotective, anti-inflammatory, and antioxidant effects and is used to treat chronic diseases, such as diabetes, liver injury, and hepatitis [25]. A study conducted by Wang et al. found that OA decreased the glucose content in the culture medium, improved insulin resistance, protected beta-cell, and inhibited the mitochondrial apoptosis in beta-TC3 cells [26]. de Melo et al.'s study [27] showed that OA reduced blood glucose and improved glucose tolerance in mice. Our study results showed that OA decreased the glucose content in the culture medium at variable dosage in HepG2 cells. It demonstrated that OA affected the glucose utilization and attenuated insulin resistance in dose-dependent manner (Figure 2). Consistence with our study, Teodoro et al. [28] found that OA enhanced insulin secretion and increased the glucose utilization in pancreatic beta-cells in vitro.

High levels of FFA could induce insulin resistance as well as inflammation [29]. Much research has shown that OA had an anti-inflammatory effect and reduced the content of inflammatory cytokines, such as TNF-*α* and IL-6. Chai et al. found that OA decreased the level of IL-6 and TNF-*α* in the serum and liver of db/db mice [30]. Liu et al. showed that OA could inhibit the generation of the inflammatory factors TNF-*α* and IL-6 [31]. Nkeh-Chungag et al. found that OA exerted potent anti-inflammatory effects by inhibiting NO and PGE2 in RAW 264.7 cells [32]. Our results showed that OA decreased the levels of IL-6 and TNF-*α* in insulin-resistant HepG2 cells (Figure 3).

Mounting evidence has suggested that inflammatory processes were related to the pathogenesis of insulin resistance [33]. The NF-*κ*B is a pivotal molecular mediator of insulin resistance [34]. Various inflammatory cytokines, including IL-6 and TNF-*α*, have been shown to activate NF-*κ*B to cause insulin resistance [35]. Insulin resistance causes the impairment of IRS1 and GLUT4, which lead to obstacle of glucose utilization [36]. As mentioned above, OA has anti-inflammatory effects. It not only reduced the content of inflammatory cytokines but also reduced the expression of NF-*κ*B and upregulated IRS1 protein expression. Kim et al. found that OA could disturb NF-*κ*B activation in 3T3-L1 adipocytes by inhibiting inflammatory responses during adipocyte differentiation through blocking IL-6-TRAF6-NF-*κ*B signaling [37]. Li et al.'s results showed that OA could upregulate IRS1 protein expression in adipose tissue in insulin-resistant rats. Our studies showed that insulin resistance induced by sodium oleate could lead to overexpression of NF-*κ*B protein and that IRS1 and GLUT4 proteins were impaired. After treatment with OA, the expression of NF-*κ*B protein was significantly reduced and the expression of IRS1 and GLUT4 protein was partially restored.

Pyrrolidine dithiocarbamate (PDTC) is a specific NF-*κ*B inhibitor that becomes widely used [38]. In Zheng et al.'s study, PDTC was used to block NF-*κ*B to explain the protective effects of chronic resveratrol treatment on vascular inflammatory injury in streptozotocin-induced type 2 diabetic rats [39]. To illustrate the point that OA could relieve the expression of the IRS1 and GLUT4 by blocking the expression of NF-*κ*B, PDTC was used to block NF-*κ*B in our study. We found that the expression of IRS1 protein in the cells of the IR group that were previously blocked by PDTC was significantly elevated compared with IR group cells without PDTC. After the addition of OA at three dosages, the expression of IRS1 protein in the OA groups was significantly higher than those in the IR group. The expression of IRS1 protein in the OA groups with PDTC was not increased as obviously as the OA groups that were not exposed to PDTC. Therefore, we thought that NF-*κ*B was the potential key target of OA that relieved insulin resistance. According to the results of Figure 4, OA could reduce the expression of NF-*κ*B protein and directly increase the expression of IRS1. As mentioned above, PDTC is a specific NF-*κ*B inhibitor. Meanwhile, the expression of IRS1 would increase when NF-*κ*B was blocked. Based on the above knowledge, we speculated that OA can relieve insulin resistance by directly affecting the expression of IRS1 protein. OA also affected the expression of IRS1 protein indirectly by regulating NF-*κ*B. When NF-*κ*B was blocked by PDTC, OA could not affect IRS1 by NF-*κ*B. Thus, the effect of OA on the expression of IRS1 protein in cells was attenuated compared to those without PDTC.

## 5. Conclusions

In conclusion, our study indicated that OA could decrease insulin resistance by reducing the content of inflammatory cytokines in culture medium. Regulating the IRS1-GLUT4 pathway via NF-*κ*B was the underlying mechanism of the effects of OA on insulin resistance. Our findings may provide new insights into the mechanisms underlying the effect of oleanolic acid in insulin-resistant cells.

## Abbreviations

IR: Insulin resistance
OA: Oleanolic acid
FFA: Free fatty acid
TNF-*α*: Tumor necrosis factor-*α*
IL-6: Interleukin-6
NF-*κ*B: Nuclear factor kappa B
IRS1: Insulin receptor substrate 1
GLUT4: Glucose transporter 4
PDTC: Pyrrolidine dithiocarbamate
ELISA: Enzyme-linked immunosorbent assay
RSG: Rosiglitazone
DMEM: Dulbecco's modified Eagle's medium
FBS: Fetal bovine serum
DMSO: Dimethyl sulfoxide.
